# The effect of semelil (angipars®) on bone resorption and bone formation markers in type 2 diabetic patients

**DOI:** 10.1186/2008-2231-20-84

**Published:** 2012-12-03

**Authors:** Shirin Hasani-Ranjbar, Zahra Jouyandeh, Mostafa Qorbani, Mahbubeh Hemmatabadi, Bagher Larijani

**Affiliations:** 1 Endocrinology and Metabolism Research Institute, Tehran University of Medical Sciences, Tehran, Iran

**Keywords:** Diabetes mellitus type 2, Bone turnover markers, Angipars®, Semelil

## Abstract

**Background and purpose of the study:**

Diabetes mellitus has been recognized as a major risk factor for osteoporosis in which bone turnover is affected by different mechanisms. As the morbidity, mortality and financial cost related to osteoporosis are expected to rise in Iran in coming years, and considering the efficacy of Angipars® for improvement of different ulcers which made it a new herbal drug in diabetic foot ulcer, there is a need to evaluate the effect of this new drug on different organs including bone resorption and bone formation markers.

**Methods:**

In this randomized, double- blind clinical trial, 61 diabetic patients were included. The subjects were randomly divided into intervention and control groups. Subjects of intervention group received 100 mg of Angipars® twice a day. Laboratory tests including bone resorption and bone formation markers were performed at baseline and after 3 months.

**Result:**

31 patients in study group and 30 patients in control group finished the study. The mean age of the study population and the mean disease duration was respectively 51.8 ± 6.2 and 7.5 ± 4.7 years with no significant differences between intervention and control patients. No statistically significant differences between patients and controls were observed in pyridinoline, osteocalcin, urine calcium, bone alkaline phosphatase and tumor necrosis factor (TNF-α). Only urine creatinine level significantly changed between two groups after 3 month of treatment (p-value: 0.029)

**Conclusion:**

In conclusion, the findings of this study indicate that Semelil (Angipars®) had no beneficial or harmful effects on bone. It might be other effects of this new component on bone turnover process which need more studies and more time to be discovered.

## Introduction

Diabetes mellitus affects skeletal system and bone metabolism through multiple pathways and it has been recognized as a major risk factor for osteoporosis [1]. Besides, osteoporosis is the most prevalent metabolic bone disease, which dramatically decreases the quality of life and increase financial burden on diabetic patients [2,3].

The relationship between diabetes and osteoporosis has been the main subject of many researches. Several mechanisms have been proposed for diabetes-related osteoporosis. Clinical trials uniformly support the fact that new bone formation and bone quality, are altered in both types of diabetes. Bone quality changes may be affected by micro vascular events common in diabetes [4,5]. Poor glycemic control can induce hypercalciuria which has been considered a potential risk factor for osteoporosis in patients with type 2 diabetes [6,7]. Besides, vitamin D deficiency is common in patients with type 2 diabetes. In the study by Dobnig et al., mean serum parathyroid hormone (PTH) and osteocalcin levels were significantly lower in treated type 2 diabetic patients; therefore, bone formation appears to be decreased in diabetes [8]. It is shown that bone metabolism and glucose metabolism are associated with each other through the action of osteocalcin [9]. Also, an increase in inflammation and associated cytokines accelerate bone turnover and bone loss in diabetic patients [4].

On the other hand, diabetes mellitus is associated with oxidative reactions. Many studies showed that increase in free oxygen radicals could induce tissue damage and abnormalities in anti-oxidant defenses in diabetes. Oxidative stress can impair the balance between proteolytic enzymes and their inhibitors that may cause or worsen osteoporosis in diabetic patients [10]. In vitro studies have shown that oxidative stress inhibits osteoblastic differentiation and induces osteoblast insults and apoptosis. Thus, oxidative stress may be related to the pathogenesis of diabetic bone disorders [11-13].

*Melilotus officinalis* (yellow sweet clover) is a species of a plant in the family of *Feabaceae*, native to Eurasia and introduced in North America, Africa and Australia. *Melilotus officinalis* contains coumarin, flavonoides (kampeferol, quercetin glycosides), triterpene saponins and volatile oil. *Melilotus officinalis* has antiphlogistic and antiedematous effects, which explain its use for inflammatory and congestive edema. It increases venous reflux and improves lymphatic kinetics [14]. Recent studies have reported a potential usefulness of administration of *Melilotus officinalis* extract for microcirculation improvement and anti-inflammation effects. Current studies showed that *Melilotus officinalis* (Angipars®) can improve diabetic foot ulcers [15,16]. *This product* also decreased nitric oxide (NO) synthesis [17] which has important effects on bone cell function and plays an important role in cytokine and inflammation induced bone loss [18]. Also it has been shown that coumarin and vitamin-K supplementations increase the serum markers for bone formation (including osteocalcin and bone alkaline phosphatase) and may reduce urinary calcium and hydroxyproline excretion (well-known markers for bone resorption). These supplements are two main components of *M*. *Officinalis* extracts [19]. As the morbidity, mortality and financial cost related to osteoporosis are expected to rise in Iran in coming years, and considering the efficacy of Angipars® for improvement of different ulcers which made it a new herbal drug in diabetic foot ulcer, there is a need to evaluate the effect of this new drug on different organs including bone. Since there is a need for alternative and complementary pharmacotherapy which has better results and less complications comparing to previous methods and considering the mechanisms involved in diabetes-induced osteoporosis [2,20,21], we decided to conduct a randomized clinical trial to examine the effect of *Melilotus officinalis* extract on bone formation and resorption markers in diabetic patients with osteoporosis.

## Materials and methods

A double blind randomized clinical trial was conducted on diabetic patients referred to the Shariati hospital diabetes clinic of the Tehran University of Medical Sciences (TUMS). These patients had previously confirmed diabetes type 2, according to the ADA (American Diabetes Association) criteria. The Participants were enrolled to the study based on the following inclusion and exclusion criteria.

Sixty one diabetic patients, between 40–60 years old, under treatment with life style modification and/or oral hypoglycemic agents and no recent inflammatory and infectious process (at least for the past 4–6 weeks and during the study) were included. Patients with chronic disease such as chronic renal failure (serum creatinine ® 270 μmol/L), chronic liver failure and decompensate heart failure were excluded. Other exclusion criteria include: coronary artery bypass graft (CABG), respiratory infectious disease, proliferative retinopathy, pregnant or lactating females, childbearing- aged women without safe contraception methods, diabetic foot ulceration or gangrene, smoking, alcohol abuse, hemoglobin a1c (HgA1C) ® 9%, any type of known drug hypersensitivity, malignancy in breast, liver and genital system, vascular disease, fracture history during the past 3 years and use of multivitamin supplements and other traditional drugs in the previous 4–6 weeks, the use of medications that affect bone metabolism, such as corticosteroids, gonadotropin releasing hormone (GnRH) analogous drugs, anticonvulsant, heparin, aluminum-containing antacids, thyroid hormone, thiazides and supplements containing calcium and other minerals. All patients participating in this study were under a same lifestyle and were matched for the drug they used; and also all the patients received just oral agents (metformin/glybenclamide).

Patients’ inclusion was completely informative and voluntary. After inclusion of each patient, complete explanation was given about the goal of study, probable side effects of the drug and patients’ rights during the research process. Then, written informed consent according to institutional guidelines was obtained before treatment. The study protocol was approved by the Medical Ethics Committee of Medical Sciences/TUMS (code number: 0011).

In this study, sample randomization was based on “Permuted Balanced Block” method by an epidemiologist. Patients were randomly assigned into 2 groups: intervention (n = 31) and control (n = 30). Semelil (Angipars®) was prepared by Pars Roos drug company (Tehran, Iran). In the intervention group, patients received 100 mg of Angipars® capsules twice a day. In the control group, participants received placebo (a non absorbable polymer) with the same dose. All participants were given a similar lifestyle program. The patients were followed for a period of three months.

For the primary assessment, a complete set of evaluations was performed which included:

1. A complete past medical history of the trial patients and an extensive physical examination were done by a trained physician, a dietary questionnaire completed at the beginning and the end of the study.

2. Measurement of bone mineral density and reporting T-score and Z-score at the baseline, to be sure if there is a major difference between patients in groups.

3. Approximately 20 ml blood (after fasting for over 12 hours) was taken from every participant. Blood samples were centrifuged within 3 hours of sampling for 20 minute to obtain serum. Samples were frozen at −80°C and they were immediately sent to the hormone laboratory of Endocrinology and Metabolism Research Institute (EMRI)/ Shariati hospital/ TUMS. Baseline laboratory tests were including a complete blood count, fasting blood sugar, HbA1C, bone alkaline phosphatase, osteocalcin, serum TNF-α, urine calcium and creatinine, urine pyridinoline.

4. Monthly documentation of patient’s compliance to therapy and acceptance of side effects.

5. Recording any probable side effects and necessary managements.

The distribution of qualitative variables was assessed by Kolmogorov-Smirnov test. Descriptive statistics, including mean, median frequencies, and percentages, were used to describe the population of study. Mean-Whitney U or *T* test and Chi-square test was performed for comparing quantitative and qualitative variables between groups respectively. The Analysis of covariance (ANCOVA) test was used for comparison outcomes between case and control group. Data were analyzed with SPSS software version 16 (SPSS Inc, Chicago, Illinois). P-values < 0.05 (2-sided test) were considered statistically significant.

## Results

Approximately eighty percent (78.7%) of participants were women. The mean age of participants in intervention and placebo group was respectively 51.87 ± 6.21 and 51.43 ± 5.37 years. The baseline characteristics of the participants are shown in Table 1. There was no significant difference in baseline characteristics and bone markers’ level between group A and B. After 3 months of treatment, no statistically significant differences between patients and controls were observed in pyridinoline, osteocalcin, urine calcium, bone alkaline phosphatase and TNF- α. Only urine creatinine level significantly changed between two groups after 3 months of treatment (p-value: 0.029). Table 2 shows mean levels of bone markers before, after 3 months and difference values between and within groups in intervention and placebo groups.

**Table 1:** Baseline characteristics of subjects in mean ± SD

**Variable**	**Group A**	**Group B**	**P-value**
**Age (yr)**	51.87 ± 6.21	51.43 ± 5.37	0.654
**BMI (kg/m2)**	28.22 ± 3.97	29.98 ± 5.32	0.269
**Lumbar T-score**	−0.36 ± 1.04	−0.25 ± 1.39	0.731
**Femoral T-score**	0.27 ± 0.97	0.21 ± 1.09	0.833
**Fasting blood sugar**	166.51 ± 51.97	180.80 ± 74.99	0.148
**Duration of diabetes**	6 (8)	8.5 (6.5)	0.154
**Sex (female)**	25 (80.6%)	23 (76.7%)	0.762

Group A: Diabetic patients on drug.

Group B: Control group.

BMI: Body Mass Index.

Duration of diabetes is presented as median (IQR).

IQR: 75^th^ – 25^th^.

Sex is presented as a number (percent).

**Table 2:** Mean levels of bone markers before and after 3 month of treatment in drug (A) and placebo (B) groups

**Variables**	**Group A**			**Group B**			**Difference**		**P-value
	**Baseline**	**3**^**rd**^**month**	*P-value	**Baseline**	**3**^**rd**^**month**	*P-value	**Group A**	**Group B**	
Pyridinoline	51.17 ± 87.90	13.06 ± 5.81	0.766	41.01 ± 38.94	15.67 ± 11.05	0.268	−40.8 ± 96.5	−28.2 ± 42.6	0.656
Osteocalcin	2.20 ± 0.47	2.5 ± 0.58	0.564	2.22 ± 0.60	2.24 ± 0.82	0.230	0.37 ± 0.80	0.23 ± 0.96	0.390
Urine Calcium	9.09 ± 9.08	8.10 ± 6.98	0.045	6.95 ± 5.03	5.77 ± 6.31	0.044	−1 ± 9.1	0.21 ± 5.9	0.683
Urine Creatinine	120.55 ± 58.59	108.23 ± 48.99	0.273	111.68 ± 72.39	130.39 ± 68.47	0.003	−11.2 ± 64	34.6 ± 49.2	0.029
Bone Alkaline phosphatase	24.9 ± 7	20.8 ± 19.7	0.014	23.8 ± 10.4	20.6 ± 7.5	0.048	−4.5 ± 7	−3 ± 10.8	0.364
TNF-α	91.68 ± 52.92	88.84 ± 55.75	0.006	90.85 ± 27.21	80.94 ± 28.73	0.026	−4.2 ± 26.1	−12.4 ± 28.2	0.267

Group A: Diabetic patients on drug.

Group B: Control group.

*P-value according to paired *T*-test.

** P-value according to ANCOVA test.

## Discussion

There are different hypothesis about the mechanisms of diabetic bone disorders including oxidative stress [11]. Increasing number of osteoporotic patients in recent years especially as a complication of diabetes mellitus causes a need for an alternative new pharmacotherapy which has better results and less complications comparing to previous methods [2]. Angipars® (Semelil) a new herbal drug which is derived from a plant named *Melilotus Officinalis* has been investigated as a useful new drug in treatment of diabetic ulcers in a clinical trial study in Iran [22].

*Melilotus Officinalis* has been proved to be helpful in reducing inflammation, regulation of the immune system and improvement of vascular blood flow which is thought to be due to the inhibitory effect of natural coumarin on oxidative DNA damage and formation [23,24]. No previously human or animal study has been published on the effects of Angipars® on bone markers in diabetic patients. Although Leilei Bao et al. found that some components in *M*. *Officinalis* were potent inhibitors of osteoclastogenesis which leads to decrease in the resorption capacity of osteoclasts [25], in this study no significant changes were seen in bone markers due to treatment with Angipars®.

Angipars® is a new herbal drug proved being useful in healing diabetic foot ulcers however the exact mechanism of its action is unknown yet. Our study showed that this drug had no adverse effect or beneficial effect on bone formation and bone resorption in diabetic patients.

Actually this study has some limitations including small sample size and short length of the study. It seems to us that a new study should be designed for another 6 months and a higher dose of Angipars® to find out whether it gives better results.

## Conclusion

In conclusion, the findings of this study indicate that Angipars® had no beneficial or harmful effects on bone. It might be other effects of this new component on bone turnover process and markers which need more studies and more time to be discovered.

## Competing interests

The authors declare that they have no competing interests.

## Authors’ contributions

Dr SR: Suggesting the main idea of the clinical trial study, preparing the proposal and editing the article (20%). Dr ZJ: Writing the article and abstract (20%). Dr MG: Epidemiologic review and data analysis (20%). Dr MH: Running the study as data collection and other performing process during the study (20%). Dr BL: Main supporter of the study (20%). All authors read and approve the final manuscript.
